# Advancing DIEP Flap Monitoring with Optical Imaging Techniques: A Narrative Review

**DOI:** 10.3390/s24144457

**Published:** 2024-07-10

**Authors:** Hailey Hwiram Kim, In-Seok Song, Richard Jaepyeong Cha

**Affiliations:** 1 Sheikh Zayed Institute for Pediatric Surgical Innovation, Children’s National Hospital, Washington, DC 20010, USA; kimpicole@gmail.com (H.H.K.); jcha2@childrensnational.org (R.J.C.); 2 Department of Oral & Maxillofacial Surgery, Korea University Anam Hospital, 73 Goryeodae-ro, Seongbuk-gu, Seoul 02841, Republic of Korea; 3 Department of Pediatrics, The George Washington University School of Medicine and Health Sciences, Washington, DC 20052, USA

**Keywords:** DIEP flap reconstruction, tissue perfusion, surgical outcomes

## Abstract

Objectives: This review aims to explore recent advancements in optical imaging techniques for monitoring the viability of Deep Inferior Epigastric Perforator (DIEP) flap reconstruction. The objectives include highlighting the principles, applications, and clinical utility of optical imaging modalities such as near-infrared spectroscopy (NIRS), indocyanine green (ICG) fluorescence angiography, laser speckle contrast imaging (LSCI), hyperspectral imaging (HSI), dynamic infrared thermography (DIRT), and short-wave infrared thermography (SWIR) in assessing tissue perfusion and oxygenation. Additionally, this review aims to discuss the potential of these techniques in enhancing surgical outcomes by enabling timely intervention in cases of compromised flap perfusion. Materials and Methods: A comprehensive literature review was conducted to identify studies focusing on optical imaging techniques for monitoring DIEP flap viability. We searched PubMed, MEDLINE, and relevant databases, including Google Scholar, Web of Science, Scopus, PsycINFO, IEEE Xplore, and ProQuest Dissertations & Theses, among others, using specific keywords related to optical imaging, DIEP flap reconstruction, tissue perfusion, and surgical outcomes. This extensive search ensured we gathered comprehensive data for our analysis. Articles discussing the principles, applications, and clinical use of NIRS, ICG fluorescence angiography, LSCI, HSI, DIRT, and SWIR in DIEP flap monitoring were selected for inclusion. Data regarding the techniques’ effectiveness, advantages, limitations, and potential impact on surgical decision-making were extracted and synthesized. Results: Optical imaging modalities, including NIRS, ICG fluorescence angiography, LSCI, HSI, DIRT, and SWIR offer a non- or minimal-invasive, real-time assessment of tissue perfusion and oxygenation in DIEP flap reconstruction. These techniques provide objective and quantitative data, enabling surgeons to monitor flap viability accurately. Studies have demonstrated the effectiveness of optical imaging in detecting compromised perfusion and facilitating timely intervention, thereby reducing the risk of flap complications such as partial or total loss. Furthermore, optical imaging modalities have shown promise in improving surgical outcomes by guiding intraoperative decision-making and optimizing patient care. Conclusions: Recent advancements in optical imaging techniques present valuable tools for monitoring the viability of DIEP flap reconstruction. NIRS, ICG fluorescence angiography, LSCI, HSI, DIRT, and SWIR offer a non- or minimal-invasive, real-time assessment of tissue perfusion and oxygenation, enabling accurate evaluation of flap viability. These modalities have the potential to enhance surgical outcomes by facilitating timely intervention in cases of compromised perfusion, thereby reducing the risk of flap complications. Incorporating optical imaging into clinical practice can provide surgeons with objective and quantitative data, assisting in informed decision-making for optimal patient care in DIEP flap reconstruction surgeries.

## 1. Introduction

The Deep Inferior Epigastric Perforator (DIEP) flap breast reconstruction has emerged as a gold standard in the field of plastic and reconstructive surgery. This innovative technique offers patients a natural, long-lasting solution for breast reconstruction by utilizing their own tissue, minimizing the complications associated with traditional implants. However, the success of the DIEP flap procedure heavily relies on the precise monitoring of flap perfusion to ensure graft viability. Optical imaging techniques have rapidly evolved, becoming indispensable tools for the real-time assessment of flap vascularization and overall tissue health [1,2,3].

Over the past few decades, optical imaging methods have gained increasing attention in the context of reconstructive surgery. These non- or minimal-invasive and real-time imaging modalities have the potential to revolutionize the postoperative monitoring of DIEP flap patients. While traditional methods for monitoring flap perfusion, such as clinical assessment and handheld Doppler ultrasound, remain fundamental, optical imaging offers significant advantages. Optical imaging methods, including near-infrared spectroscopy, laser speckle contrast imaging, indocyanine green angiography, and hyperspectral imaging, provide detailed insights into tissue oxygenation, perfusion, and overall tissue vitality.

This review paper aims to provide an extensive and up-to-date overview of the various optical imaging techniques employed for monitoring DIEP flap procedures. We will explore the principles, methodologies, and clinical applications of these techniques in detail, shedding light on their ability to enhance the safety and success of DIEP flap breast reconstruction.

The DIEP flap surgery, with its meticulous microvascular anastomosis and intricate tissue transfer, demands real-time monitoring to assess vascular compromise, enabling prompt intervention if complications arise. Optical imaging techniques offer the potential to predict and detect vascular insufficiency at an earlier stage than traditional monitoring methods, potentially reducing the need for reoperations and improving patient outcomes. By surveying the current state of research and clinical practices, this review journal will provide a comprehensive resource for plastic surgeons, researchers, and medical professionals interested in the forefront of DIEP flap monitoring.

We will delve into the strengths and limitations of each optical imaging technique, offering insights into their specificity, sensitivity, and practicality in the clinical setting. Moreover, we will examine recent advancements and emerging technologies that have the potential to further enhance the precision and ease of DIEP flap monitoring.

## 2. Materials and Methods

### Literature Review and Selection Process

**Figure 1:**
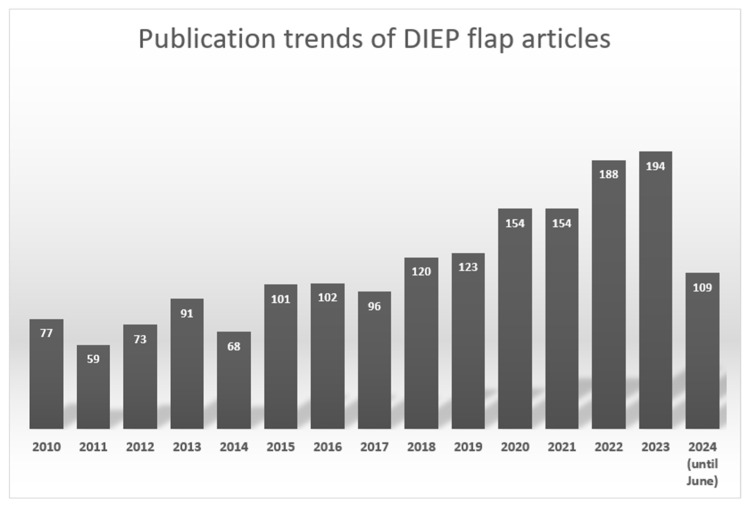
Number of articles published on DIEP flap reconstruction per year.

A comprehensive search was conducted across PubMed, MEDLINE, and relevant databases, including Google Scholar, Web of Science, Scopus, PsycINFO, IEEE Xplore, and ProQuest Dissertations & Theses, among others. We have analyzed the publication trends for DIEP flap reconstruction using PubMed data. This analysis included extracting the number of articles published each year from 2010 to 2023. The total number of articles published each year is shown in Figure 1. We conducted a comprehensive literature search using specific keywords related to optical imaging, DIEP flap reconstruction, tissue perfusion, and surgical outcomes. These keywords were carefully selected to cover all relevant aspects of our study. The detailed keywords used for each case are listed in Table 1. This extensive literature review aimed to identify studies focusing on optical imaging techniques for monitoring DIEP flap viability. Articles discussing the principles, applications, and clinical use of NIRS, ICG fluorescence angiography, LSCI, and HSI in DIEP flap monitoring were selected for inclusion. Data regarding the techniques’ effectiveness, advantages, limitations, and potential impact on surgical decision-making were extracted and synthesized.

## 3. Results

**Figure 2:**
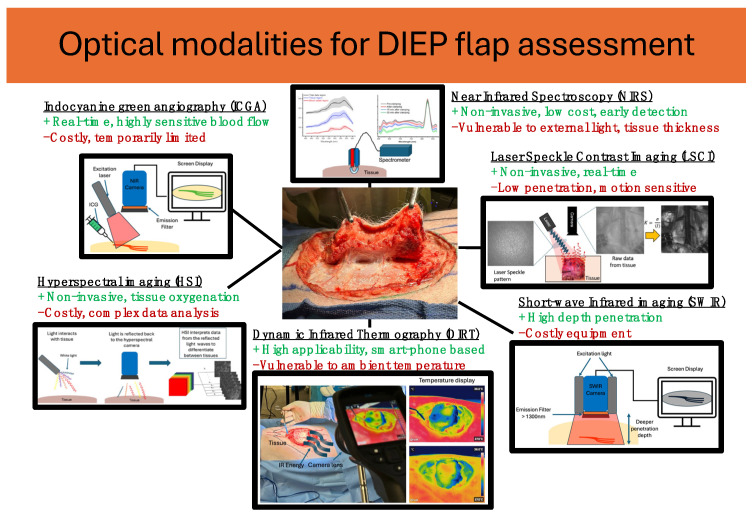
Illustration of optical imaging techniques detailing the relevant equipment during DIEP flap harvesting.

Optical imaging modalities, including NIRS, ICG fluorescence angiography, LSCI, HSI, DIRT, and SWIR offer a non- or minimal-invasive, real-time assessment of tissue perfusion and oxygenation in DIEP flap reconstruction (Figure 2).

### 3.1. Near-Infrared Spectroscopy (NIRS)

The current state of near-infrared spectroscopy (NIRS) in monitoring DIEP flap procedures, especially in breast reconstruction surgeries, underscores its growing significance and effectiveness. NIRS, a non-invasive optical method, offers continuous bedside monitoring of flap oxygenation, providing essential insights into tissue perfusion and vitality [4]. The technique works by emitting near-infrared light into tissue, which is absorbed by chromophores like oxy- and deoxyhemoglobin [5]. The reflected light is measured to ascertain the tissue’s oxygenation status. This method allows for continuous monitoring of regional tissue oxygen saturation (rSO2), which is vital for evaluating flap viability [6].

In terms of accuracy and effectiveness, NIRS has demonstrated high accuracy in flap monitoring [7,8]. Kagaya and Miyamoto’s eight extensive reviews of fifteen clinical and eight animal studies indicated a 99.5% overall success rate in flap surgeries, with a 91.1% flap salvage rate when StO2 levels were regularly measured. This method showed high sensitivity and specificity in detecting vascular issues. Newton et al. analyzed ten articles from 590 studies, highlighting NIRS’s remarkable accuracy in flap monitoring, with an 89% flap salvage rate compared to 50% with conventional methods [9]. The study also noted that NIRS detected vascular compromise significantly earlier than clinical signs, with low false positive and negative rates. Bian et al. reported that NIRS consistently showed specificity and sensitivity values above 95% and 96%, respectively, for postoperative flap monitoring [10]. This supports the potential of NIRS as an objective, accurate, and continuous monitoring technique for postoperative free flaps. Highlighting its advantages, Berthelot et al. found that NIRS was superior to conventional clinical assessments in detecting free tissue transfer failure, thus underscoring its effectiveness in breast reconstruction [11].

A systematic review of NIRS in flap monitoring revealed a 99.5% overall flap success rate, demonstrating its capability to detect vascular compromise with high sensitivity and specificity [1]. This highlights NIRS’s significant contribution to the success of breast reconstruction surgeries by facilitating early intervention in cases of compromised tissue perfusion. However, NIRS does have limitations, as its accuracy can be affected by external factors such as ambient light and tissue thickness [12]. Despite this, NIRS is non-invasive, relatively low-cost, and increasingly popular for studying cortical activation and changes in the cytochrome oxidase redox state in response to oxygen delivery alterations [13,14]. NIRS is a promising, reliable, non-invasive, and effective tool for monitoring flaps in surgeries, providing valuable data on tissue oxygenation and perfusion. Its application in breast reconstruction and exercise physiology, along with its cost-effectiveness, makes it a valuable asset in medical practice. As the technology continues to evolve, it is poised to become an integral part of postoperative monitoring and care for patients undergoing reconstructive procedures.

### 3.2. Indocyanine Green Angiography (ICG-A)

Indocyanine green angiography (ICG-A) has become a pivotal tool in monitoring DIEP flap procedures, offering significant advantages in assessing tissue oxygenation, perfusion, and overall tissue vitality. ICG-A differs from Fluorescein Angiography (FA) in its dye properties and tissue penetration capabilities. ICG-A utilizes indocyanine green dye, which is highly protein-bound and fluoresces in the infrared spectrum, allowing it to visualize deeper vascular structures like the choroidal vasculature [15]. Conversely, FA employs sodium fluorescein, which is less bound to protein and fluorescing in the visible spectrum, effectively imaging the retinal vasculature [16]. When combined, ICG-A and FA provide a comprehensive vascular assessment. ICG-A images deeper structures, and FA focuses on more superficial retinal vessels [17]. This synergistic approach is particularly valuable in complex diagnostic or surgical scenarios, where understanding superficial and deep vascular patterns is crucial.

In DIEP flap breast reconstructions, ICG-A has proven to be an accurate and reliable method for assessing problematic perfusion, significantly decreasing the rate of fat necrosis [18] and the need for reoperation [19,20]. It is beneficial in intraoperative assessments, ensuring adequate flap perfusion and viability [20]. Recognized for providing real-time information on blood flow, ICG-A informs intraoperative decisions regarding flap design and modifications [21]. ICG-based FA is a novel, useful tool for various applications in breast reconstruction [22]. ICG-A provides objective data on tissue perfusion, more accurately guiding surgical decisions than subjective clinical judgment [23]. However, the need for specialized equipment can make ICG-A more costly and less accessible in some settings. In terms of specificity and sensitivity, ICG-A is highly sensitive to changes in microvascular blood flow, making it suitable for assessing tissue perfusion in breast reconstruction surgeries [21]. For example, in the case of mastectomy flaps, the use of ICG-A has been shown to achieve 90% sensitivity and 100% specificity in reducing the risk of skin flap necrosis and lowering the overall complication rate [21]. Meanwhile, the success rate of free flaps has been reported at 92.0% [24]. Practical for intraoperative use, ICG-A offers an immediate and accurate assessment of tissue perfusion [25]. Despite its high sensitivity (90.9%) and accuracy (98.6%), the research has addressed the lack of consensus on ICG dosage and the need for pre-operative allergy testing, aiming to establish safer and more effective ICG application guidelines in flap surgeries [26]. Given the above discussions, indocyanine green angiography (ICG-A) represents a significant advancement in optical imaging techniques for monitoring DIEP flap procedures. Its ability to provide real-time, objective data on tissue perfusion is invaluable in assessing flap viability and reducing postoperative complications. Despite its limitations, ICG-A shares similarities with LSCI in being minimal-invasive and highly sensitive to microvascular changes, making it effective in breast reconstructive surgery. However, for optimal patient outcomes, clinicians should integrate ICG-A findings with other clinical assessments.

### 3.3. Laser Speckle Contrast Imaging (LSCI)

LSCI has seen significant advancements and is increasingly utilized for its ability to provide detailed insights into tissue oxygenation, perfusion, and overall tissue vitality. LSCI operates on the principle of laser speckle, a random interference pattern created when laser light scatters from a diffusing surface [27]. In biological tissues, the movement of red blood cells blurs this speckle pattern, quantifiably indicating blood flow [28]. LSCI uses a laser to illuminate the tissue of interest, capturing the speckle pattern with a camera. This blurring is then analyzed, quantitatively measuring blood flow or perfusion in the tissue.

Clinically, LSCI excels in providing real-time, high-resolution images of microvascular blood flow, crucial for assessing DIEP flap viability during and after reconstructive surgery [29]. It is particularly effective in identifying areas at risk of necrosis due to poor perfusion. They demonstrated LSCI’s potential in predicting flap necrosis, finding that decreased perfusion immediately post-surgery and perfusion below 25 PU at 30 min were predictors of tissue morbidity 72 h after surgery [30]. Intraoperatively, LSCI assists in real-time assessment of perfusion adequacy, aiding surgeons in optimizing flap design and anastomosis placement.

LSCI’s strengths include non-contact, real-time tissue perfusion assessment without needing intravenous contrast agents. Also, it offers high-resolution images that allow for detailed mapping of microvascular blood flow across the tissue. However, LSCI is limited to superficial tissues [31], as its penetration depth is relatively shallow [32]. In terms of specificity and sensitivity, LSCI is highly effective in detecting changes in superficial microvascular blood flow, with its accuracy (sensitivity and specificity) often exceeding 90% [33]. However, this accuracy greatly depends on how it is used and the initial state of blood flow in the area being observed [29,34]. It is an excellent tool for monitoring the immediate perfusion status of superficial tissues like skin flaps [30]. While LSCI is practical for intraoperative use, given its real-time feedback, its utility is limited to superficial tissue analysis and may require integration with other modalities for comprehensive flap monitoring [35]. In light of the above discussions, LSCI represents a significant advancement in optical imaging techniques for monitoring DIEP flap procedures. Its capacity to provide real-time, high-resolution images of tissue perfusion is invaluable in assessing flap viability. Despite limitations in depth penetration and quantitative analysis, its non-invasive nature and sensitivity to microvascular changes make LSCI a powerful tool in reconstructive surgery. However, clinicians should integrate LSCI findings with clinical judgment and potentially other monitoring modalities for optimal patient outcomes.

### 3.4. Hyperspectral Imaging (HSI)

Hyperspectral Imaging (HSI), particularly in breast reconstruction surgeries, is an evolving field that leverages advanced imaging technologies to provide crucial insights into tissue health [36]. Recognized for its potential to transform postoperative monitoring and enhance surgical outcomes, HSI is gaining increasing recognition [37]. This technique offers a comprehensive analysis of various tissue parameters, such as tissue oxygenation (StO2%), near-infrared (NIR%) perfusion, tissue hemoglobin (THI%), and tissue water (TWI%). These parameters are pivotal in evaluating tissue vitality and ensuring flap surgery success. For example, studies have demonstrated that in DIEP flap surgeries where StO2 and NIR PI values exceeded 40, consistent healing was observed without the need for revision surgery [36,38]. HSI functions by capturing images across an extensive range of light wavelengths (Table 2) [39]. This technique enables the analysis of unique tissue characteristics, as each tissue type absorbs and reflects light differently. The processed images yield a ‘spectral fingerprint’ of the tissue, thus revealing intricate details about its health [40].

However, the complexity of interpreting hyperspectral data might necessitate specialized expertise. In the context of breast reconstructive surgery, this involves establishing definitive threshold values for adequate tissue perfusion, such as StO2 and NIR PI values above 40, and guiding surgeons in identifying optimal anastomosis sites [39]. It should be noted that HSI’s application is limited to open surgery due to the substantial size of the HSI camera. Despite these limitations, HSI’s potential in breast reconstruction is considerable. Its capacity to provide detailed, real-time information on tissue health can assist in the early detection of complications, potentially enhancing surgical outcomes and patient care [59]. This technology offers a novel, more accurate, and efficacious monitoring approach compared to traditional methods [23].

### 3.5. Thermographic Imaging and Dynamic Infrared Thermography (DIRT)

Thermographic imaging, particularly dynamic infrared thermography (DIRT), is becoming promising in monitoring Deep Inferior Epigastric Perforator (DIEP) flap procedures [60]. This non-invasive imaging technique provides valuable insights into tissue health, including aspects of oxygenation, perfusion, and overall vitality [61]. Thermographic imaging detects temperature variations on the skin surface using infrared technology, which are indicative of underlying vascular activity [17]. Areas with robust blood supply typically appear warmer, reflecting higher blood flow [62]. DIRT uses infrared cameras to capture thermal images of the surgical area to visualize perfusion in the flap, which allows for successful viability. In this way, DIRT distinguishes itself by dynamically monitoring temperature variations over time, providing surgeons with a more detailed view of blood flow and tissue health [63]. This makes DIRT especially suitable for intricate surgical procedures, providing real-time insights during DIEP flap monitoring [64].

In DIEP flap surgeries, DIRT is essential for perforator mapping, helping surgeons select the most suitable perforators to optimize surgical outcomes [60,65]. Post-surgery, thermographic imaging monitors flap viability, identifying areas at risk of compromised perfusion and potential necrosis [66]. Thermographic imaging’s primary advantage is its non-invasive nature and ability to provide real-time feedback on tissue perfusion without the need for contrast agents [62]. Its practicality stems from its non-invasive nature and ease of use, making it suitable for intraoperative and postoperative monitoring [67]. However, the technique’s efficacy can be affected by external factors such as ambient temperature, and its image resolution may not match other modalities, such as Computed Tomography Angiography (CTA) [65]. While sensitivity to changes in surface temperature correlated with blood flow, its specificity may be less than direct vascular imaging methods, such as ICG-FA, which provide clearer insights into tissue perfusion and vascular structures [68].

In breast reconstruction surgeries, thermographic techniques, such as DIRT, offer practical and efficient methods to ensure optimal flap perfusion and viability [65,69]. A clinical study involving [30] DIEP flaps in 21 patients demonstrated DIRT’s effectiveness in verifying perforator positions as indicated by CTA and overseeing flap viability during and after surgery, leading to positive outcomes [70]. The accuracy of sensitivity and specificity measurements for certain applications shows considerable variation, with some research indicating sensitivity levels reaching between 85% and 90% and specificity figures closely aligning within this range [65,71,72]. DIRT provides information on perforator location, blood supply, and anastomosis integrity without disrupting the surgical process. It has also shown promising results in preoperative perforator mapping, intraoperative perfusion assessment, and postoperative flap monitoring, proving to be safe, fast, non-invasive, cost-effective, and reliable [73]. DIRT can be an alternative to CTA for perforator mapping, as it does not require ionizing radiation or contrast injection [74]. Other clinical studies have indicated that DIRT is superior to hand-held Doppler (HHD) for detecting perforators intraoperatively [75,76]. The advancement in DIRT technology involves the development of a smartphone-compatible miniaturized thermal imaging camera [77]. Although the resolution of the affordable FLIR ONE© (FLIR Systems, Wilsonville, OR, USA) is not as high as more expensive thermal imaging cameras, it provides a convenient screening tool requiring minimal training [78]. This tool can be easily carried in a pocket, offering quick and effortless access in a clinical setting.

### 3.6. Short-Wave Infrared (SWIR) Imaging

Short-wave infrared (SWIR) imaging is emerging as an important advancement in medical imaging, with the potential for enhancing the monitoring of Deep Inferior Epigastric Perforator (DIEP) flap procedures. Utilizing the short-wave segment of the infrared spectrum, which ranges from approximately 1.4 to 3 μm [79], SWIR imaging is distinguished by its ability to produce images through the differential absorption and reflection of SWIR light by biological tissues [80]. This can be achieved by leveraging the unique spectral signatures that different tissue types exhibit within the SWIR range. A key advantage of SWIR light is its capability to penetrate deeper into tissues than visible light, with significantly reduced absorption and scattering [81]. This feature enables the clear visualization of subcutaneous structures, providing an in-depth view beneath the skin surface without the need for invasive methods [82,83].

Despite its potential, the clinical implementation of SWIR technologies in DIEP flap surgery remains limited [80]. Nevertheless, SWIR imaging shows promise in various crucial aspects, such as identifying and evaluating the viability of perforator vessels, which are essential for the success of the surgery [56]. By generating detailed images that display blood flow and oxygenation levels, SWIR imaging can facilitate intraoperative and postoperative monitoring of the DIEP flap’s perfusion, thus ensuring its viability [81]. Furthermore, this modality provides the benefit of early detection of potential flap complications, such as compromised blood flow or tissue necrosis, enabling timely interventions [56]. The use of SWIR imaging can significantly enhance surgical planning and outcomes, potentially improving surgical results and reducing the risk of flap failure by allowing for the visualization and selection of optimal perforators [61]. As a non-invasive method, SWIR imaging offers a patient-friendly solution for continuous flap health monitoring, eliminating the need for direct contact or invasive probes [56,80]. Furthermore, SWIR can be integrated with machine learning (ML), enhancing precision in disease detection and treatment guidance and moving towards a more data-driven approach to personalized healthcare [84].

However, there are challenges to adopting SWIR thermographic imaging for DIEP flap monitoring, including the need for specialized and potentially costly equipment and the requirement for expertise to accurately interpret SWIR images, which may restrict its use to well-equipped centers [81,82]. Moreover, the potential of SWIR imaging to detect small-vessel structures, critical for diagnosing complex vascular conditions such as hemangiomas and congenital vascular malformations, remains underexplored [85,86]. Furthermore, while the enhanced visualization of subcutaneous structures is a considerable benefit, the penetration depth of SWIR light may not meet the needs for imaging deeper tissue structures [87]. This highlights the importance of ongoing research and technological development to expand the medical applications of SWIR imaging. Integrating various optical imaging modalities can also lead to a more comprehensive understanding of flap perfusion and viability, enhancing the potential of SWIR imaging in clinical settings.

The diverse characteristics of optical imaging techniques for DIEP flaps translate into distinct clinical applications. Table 3 summarizes the advantages and limitations of each modality for informed clinical decision-making.

## 4. Future Perspective

The potential of using AI in image resolution enhancement and flap perfusion diagnosis in medical imaging and surgery is promising because these advancements can significantly improve patient outcomes, diagnostic accuracy, and the efficiency of medical procedures [88,89]. Emerging studies highlight the evolution of preoperative planning in autologous breast reconstruction, focusing on the shift from traditional ultrasound to advanced 3D imaging techniques and the integration of artificial intelligence, machine learning, and augmented reality to overcome the challenges of complex vascular anatomy in DIEP flap procedures [69,90]. The application of AI, particularly deep learning techniques, in enhancing the resolution of medical images has shown promising results. For example, Convolutional Neural Networks (CNNs), a type of deep learning technique, have become very successful and widely used for analyzing images [91,92]. Deep learning can also turn a regular RGB image, which only has three color channels, into a multi- or hyperspectral image that contains much more detailed spectral information [93]. This is possible because recent advancements in deep learning have taught AI models to understand and expand on the spectral data initially captured in an RGB image. In the context of flap perfusion diagnosis, AI technologies also offer the potential to understand, predict, and monitor the viability of transplanted tissues. For example, techniques from computer vision and machine learning can be used to analyze how a fluorescent dye called ICG moves into and out of tissues in real-time [6]. This analysis allows researchers to mathematically predict certain tissue behaviors. By examining these behaviors, it is possible to classify them into categories that are important for clinical purposes. This means that AI can help us understand the characteristics of different tissues based on how they interact with ICG. Furthermore, mobile smartphone thermal imaging (MTI) can be a fast, precise, and simple way to collect data that can detect issues in very small surgical connections [7]. This approach is highly promising as a support tool for surgeons in the preparation, execution, and follow-up care of DIEAP flap surgeries. However, it is important to note that the integration of AI into clinical practice is not without challenges. Issues such as data privacy, ethical considerations, and the need for robust validation of AI algorithms must be addressed [88]. Moreover, adopting AI technologies requires significant training for healthcare professionals, ensuring they can effectively interpret AI-enhanced images and make informed clinical decisions. The future of AI in medical imaging and surgery is bright, with ongoing research and technological advancements.

## 5. Conclusions

In conclusion, optical imaging techniques represent a promising frontier in the evolution of DIEP flap surgery. This review journal aims to consolidate the existing knowledge and highlight the potential of these optical imaging techniques in the context of breast reconstruction. While challenges remain, such as penetration depth limitations, technical sensitivity, and environmental vulnerability, ongoing research strives towards more precise and indication-specific applications that will ensure consistent and reliable image acquisition. By offering a comprehensive understanding of the current landscape and future prospects, it will serve as a valuable resource for the medical community working towards optimizing the outcomes of DIEP flap procedures.

## Figures and Tables

**Table 1:** Keywords used in literature search.

Case	Keywords
Optical imaging	“optical imaging”, “imaging techniques”, “non-invasive imaging”, “optical coherence tomography”, “fluorescence imaging”, “near-infrared spectroscopy”, “hyperspectral imaging”, “laser speckle contrast imaging”
DIEP flap reconstruction	“DIEP flap”, “deep inferior epigastric perforator flap”, “breast reconstruction”, “flap surgery”, “microsurgery”, “tissue transfer”
Tissue perfusion	“tissue perfusion”, “blood flow”, “microcirculation”, “perfusion imaging”
Surgical outcomes	“surgical outcomes”, “postoperative results”, “surgery success rates”, “complication rates”

**Table 2:** Optical wavelengths utilized in each modality.

Optical Modality	Wavelength Used	References
Near-infrared spectroscopy (NIRS)	700–1000 nm	[19,41,42,43,44]
Indocyanine green (ICG fluorescence angiography)	750–810 nm	[17,19,23,45]
Laser speckle contrast imaging (LSCI)	660–785 nm	[30,46,47,48]
Hyperspectral imaging (HSI)	400–1000 nm	[38,49,50,51]
Dynamic thermography (DIRT)	650–1400 nm	[52,53,54,55]
Short-wave infrared thermography (SWIR)	1000–2500 nm	[56,57,58]

**Table 3:** A summary of the intraoperative imaging modalities during DIEP harvesting.

Modality	Concept Description	Advantages	Limitations
Near-infrared spectroscopy	Analyzes tissue by measuring how it absorbs near-infrared light	Noninvasive; low-cost; detects vascular compromise early and fast	Its accuracy can be affected by external factors such as ambient light and tissue thickness
Indocyanine green angiography *	Utilizes indocyanine green dye allowing it to visualize deeper vascular structures like the choroidal vasculature	Real-time; highly sensitive and specific to blood flow	Costly; minimally invasive; safety issue (allergy)
Laser speckle contrast imaging	A random interference pattern is created when laser light scatters from a diffusing surface of red blood cells	Noninvasive; real-time	Low penetration depth; technique sensitive
Hyperspectral imaging	Hyperspectral imaging captures and analyzes light across a very wide range of wavelengths, creating a detailed “fingerprint” of an object’s composition.	Noninvasive; real-time; comprehensive analysis possible	Complex data interpretation
Thermographic imaging and dynamic infrared thermography	Detects temperature variations on the skin surface using infrared technology, which are indicative of underlying vascular activity	Noninvasive; real-time; high applicability (smartphone)	Vulnerable to ambient temperature; low image resolution
Short-wave infrared imaging	Uses light beyond the visible spectrum to create detailed heat maps of objects, revealing temperature variations not seen with the naked eye	Noninvasive; real-time; deeper penetration than visible light;	Costly; difficulty in data interpretation

* Gold standard intraoperative perfusion monitoring technique for DIEP flaps.
